# Rheological Evaluation of Softened Binders Blended with Aged Asphalt Selected with a High-Temperature Mixing Chart

**DOI:** 10.3390/ma15051775

**Published:** 2022-02-26

**Authors:** Leonardo Ochoa-Ambriz, María de la Luz Pérez-Rea, Genoveva Hernández-Padrón, Pedro Limón-Covarrubias, José Roberto Galaviz-González, David Avalos-Cueva

**Affiliations:** 1 Faculty of Engineering, Autonomous University of Queretaro, Santiago de Querétaro 76010, Mexico; leonardo8a92@hotmail.com (L.O.-A.); perea@uaq.mx (M.d.l.L.P.-R.); 2 Department of Nanotechnology, Centro de Física Aplicada y Tecnológia Avanzada, Universidad Nacional Autónoma de México, Santiago de Querétaro 26230, Mexico; genoveva@fata.unam.mx; 3 Department of Civil Engineering and Topography, University of Guadalajara, 1421 Blvd. Marcelino García Barragán, Guadalajara 44430, Mexico; ingenieria_limon@hotmail.com (P.L.-C.); jose.galaviz2401@academicos.udg.mx (J.R.G.-G.)

**Keywords:** blending chart mix, linear amplitude sweep, multiple stress creep recovery, reclaimed asphalt pavement

## Abstract

Reclaimed asphalt pavements (RAP) provide economic and environmental benefits. In recent decades, their use has increased, but rheological properties are affected by RAP aging, increasing stiffness, cracking, and susceptibility to water. To counteract these effects, rejuvenating agents are used, but they must be properly dosed to design quality mixtures. Therefore, different binders were analyzed, including virgin binder (VBB), binder modified by SBS polymer (MB), AC-RAP, binder softened using a rejuvenating agent, and binders softened with doses (15%, 30%, and 45%) of AC-RAP. The rheological properties were evaluated by dynamic shear rheometry (DSR) and beam-bending rheometry (BBR) tests, while the linear amplitude sweep (LAS) test was used to measure fatigue cracking and the multiple stress creep recovery (MSCR) test was used to measure rutting. A mixing chart was constructed based on a high temperature AC-RAP to satisfy the performance grade (PG 76-22). The results showed that softened binders become flexible, but when AC-RAP is added, they turn stiff and behave better than MB. Moreover, combining a rejuvenating agent and AC-RAP reduces the aging stiffness of RAP, improving its rheological properties without compromising the rutting or cracking resistance.

## 1. Introduction

The pavement industry is a vital sector that can actively serve a country’s economy by contributing to the GDP [1], although it has grown tremendously, leading to shortages and higher prices for aggregates and binder. [2]. Just 360 million metric tons of asphalt pavement are produced annually in the U.S. [3], being the world’s most recycled building material, which can be processed and used to replace virgin aggregates and binders [4,5,6]. According to the Federal Highway Administration (FHWA), approximately 100 million tons of hot mix asphalt (HMA) are produced each year [7,8,9], as it is the most widely used paving material in the world [4,10]. Thus, the road sector is moving towards sustainable development, as it aims to reduce the consumption of nonrenewable resources [5,11,12] through reclaimed asphalt pavements (RAP) [2]. These are removed materials, consisting of granular pieces of milled asphalt pavement [3,10] of high quality and well graded [9]. RAP can be extracted by central plant hot recycling (CPHR), hot in place recycling (HIR), central plant cold recycling (CPCR), and cold in place recycling (CIR) processes [13]. Milad, Taib, Ahmeda, Solla and Yusoff [9] explained that RAP is the most widely used method in rehabilitation and construction sites, as it is more environmentally friendly and cost-effective. From this, the European Asphalt Pavement Association (EAPA) showed that 47% of available RAP was used in hot applications, while the National Asphalt Pavement Association (NAPA) estimated that 84% was used in U.S. asphalt applications [7,11,12].

In accordance with Milad, Taib, Ahmeda, Solla and Yusoff [9], The Department of Transportation (DOT) uses 15% RAP in surface pavement layers made with HMA mixes, while in the U.S. the use of RAP ranges from 12% to 15%. NAPA proposed to increase this amount to 25% in 2013. In addition, the NCHRP project D9-12 determined that the RAP content is between 10% and 20% [5,6]. On the other hand, several studies have reported using RAP at rates between 20% and 50% [3,7,14,15,16], but some studies have demonstrated the feasibility of producing asphalt mixtures containing high (40%) or very high (100%) RAP contents [5]. An analysis of the volumetric properties and performance of asphalt mixtures was carried out by Antunes, Freire and Neves [4], which showed fourteen case studies in which RAP was used at rates of 10% to 40%, 10% to 60%, and 100%.

Consequently, if 100% of the RAP is used, the need for virgin materials would be reduced, thereby inducing sustainability in pavements [11]; hence, RAP has been widely used because it helps to reduce costs in the construction of flexible pavements, conserves natural resources [4,8,17,18,19,20], requires low amounts of energy and fuel [1,9], eliminates solid waste, and reduces the carbon footprint [3,10,11,12]. In this sense, according to Debbarma and G. D. Ransinchung [21], there were reductions of 67% and 18% in cost and gas emissions, respectively, when RAP was used in asphalt mixtures, while CO_2_ emissions were reduced by 2%.

However, the maximum quantity of RAP that can be used to produce HMA is limited [2,11], since the performance of RAP in the mixture has been questioned because it is only considered as an aggregate [22,23]. As a result, many state agencies do not permit the use of more than 40% of RAP in mixtures [10]. Therefore, there are negative perceptions and practical problems limiting the use of RAP: (a) the quality of the RAP aggregates; (b) the technology of the production plant; (c) the mix design methodology; (d) the performance of the final mix containing RAP [1,5]; (e) the normative restrictions [4]. It is now known that the interfacial binding energy between the asphalt-coated RAP aggregates causes a reduction in the resistance of the RAP in the mixtures [1,11]. In addition, if the recycled pavement is incorrectly designed, the expected service life of the pavement will be reduced [10]. In the same way, due to the inconveniences explained by Devulapalli, Kothandaraman and Sarang [8], as well as Li, et al. [24], as RAP is exposed to weathering factors such as temperature, ultraviolet light, rain, and oxygen during its useful life, the RAP binder (AC-RAP) undergoes aging, causing it to lose its rheological properties, having high viscosity (stiffness) and poor elasticity, which stiffens the asphalt mix. [1,2,10,18,19,25]. Rheology studies relate to the internal responses of viscoelastic materials such as asphalts when they are deformed due to applied stresses. This is of interest because asphalt mixtures modify their viscosity as temperature changes; at high temperatures, the binder is a viscous fluid, while at low temperatures, it is considered an elastic solid [26]. However, viscosity and penetration are sometimes not sufficient to determine asphalt performance in situ [27]. Therefore, the evaluation of the rheological properties of binders is important because it helps to predict the performance of asphalt pavements [28].

These properties have been of interest, since they directly affect the behavior and performance of asphalt mixtures [29,30,31,32]. A literature review confirmed that the stiffness of RAP blends is higher than that of virgin blends [6,33]. Low RAP levels (<50%) do not greatly affect the resistance, porosity, water absorption, reduced rutting, increased tensile strength, or modulus [1,2,3,7,9,11,15]. Additionally, as the RAP content increases, the cracking deterioration [5,8,12,34,35,36] and susceptibility to moisture increase [10], while the dynamic modulus and tensile strength decrease [15,17].

To counteract the negative effects of the use of RAP, there are methods that help reduce stiffness and increase rutting resistance, such as the use of WMA technologies, the use of a softer binder, increasing the binder dosage, or the application of rejuvenating agents [6,15,18,24,33,37,38]; however, some adjustments must be made to the mix design in order to achieve the proper mix performance [5]. Softening of reclaimed asphalt pavement is usually achieved by adding a rejuvenating agent to the aged pavement mixture, reconstituting the chemical compositions of the aged binders [10,12,20,36], although this depends on the type and dosage of rejuvenator, since overdosing will increase the flexibility of the mixtures, while underdosing will increase the stiffness [8]. The rejuvenator must satisfy both short- and long-term criteria. First, it must melt rapidly in the AC-RAP to mobilize the aged asphalt, while in the long term the chemical and rheological properties must be modified [10]. Kaseer, Bajaj, Martin, Arámbula-Mercado and Hajj [33] conducted an analysis, utilizing calculated doses of rejuvenating agents that helped decrease brittleness and increase long-term crack resistance. Wang, Zeng, Qin, Huang and Xu [36] concluded that the rejuvenating agent improves the fracture energy and fracture tenacity of recycled mixtures containing high RAP contents, although if the RAP content is high, it is difficult to soften it. On the other hand, Cheraghian and Wistuba [39] carried out an analysis of the surface morphology and rheological and chemical properties of a binder modified by a composite of clay and fumed silica nanoparticles (NPs) exposed to ultraviolet (UV) aging. They found that the surfaces of the clay nanocoatings and the high UV reflectivity of the fumed silica NPs led to changes in binder performance, increasing stiffness and improving resistance to permanent deformation. Rheological results with dynamic shear rheometer (DSR) and Fourier transform infrared spectroscopy (FT-IR) showed increased resistance to UV aging, delaying the hardening of the binder. In addition, Cheraghian and Wistuba [40] used fumed silica NPs to evaluate their effects on UV aging resistance. Rheological tests confirmed that they increased the UV aging resistance, increased the complex modulus, and the reduced stiffness due to the shielding produced by the NPs. Finally, a study conducted by Cheraghian and Wistuba [40] showed a novel development of an asphalt binder containing fumed silica NPs in HMA mixtures, reducing the temperature and moisture susceptibility. Furthermore, it can reduce the rate of viscosity aging in the short and long term.

Currently, the absolute viscosity and PG methods are used to estimate the proportion of RAP [41]. Superpave’s standard mix design method (which is specified in the standard AASHTO M323 [42]) for more than 25% RAP content based on the NCHRP 9-12 project suggests the use of a mixing chart to determine the proportion of RAP that can be added using the given binder or the grade of binder to use for the desired RAP content [6], assuming a homogeneous mix between AC-RAP and virgin asphalt, with 100% effectiveness. Thus, the AASHTO M323 [42] standard also specifies the calculation of low, intermediate, and high temperatures for selection. However, according to Singh, Showkat and Sawant [41], Superpave’s rutting parameter, G*/sinδ, which evaluates the high-temperature PG of a binder and aids in the construction of the high-temperature PG mixing chart, is considered inadequate to properly evaluate the performance of modified binders. The blending charts are based on the critical temperature of the asphalt binder, a questionable parameter regarding the performance of the mixture if the binder used contains adhesion-promoting polymers [43]. However, the actions of rejuvenating additives, as well as polymers, could change the performance of the HMA [44].

Consequently, the objective of this article is to evaluate the rheological performances of different asphalt binders, including virgin binder (VBB), RAP asphalt cement (AC-RAP), a modified binder (MB) with styrene-butadiene-styrene polymer (SBS), softened binders using the rejuvenating agent “Maro-1000^®^” (SMBL, SMBM, and SMBH), and softened binders blended with different percentages (15%, 30%, and 45%) of AC-RAP (SMBL15, SMBM30, and SMBH45, respectively), which were designed based on a high-temperature mixing chart constructed by linear characterization of the performance grade (PG). The rheological performances were measured using a dynamic shear rheometer (DSR) and a beam-bending rheometer (BBR), while through the linear amplitude sweep (LAS) and multiple stress creep recovery (MSCR) test the fatigue cracking behavior was measured. The frequency sweep accelerated the rate of damage accumulation using the continuous viscoelastic damage theory (VECD).

## 2. Materials and Methods

### 2.1. Virgin Binder Base (VBB)

The asphalt binder used in this experimental research was classified by its PG grade, 64-22, through the standardized procedure described in AASHTO M323 [42]. 

### 2.2. Modified Binder (MB)

The VBB was modified using an SBS-GP100 polymer at a proportion of 2.25% with respect to the weight of the asphalt binder. SBS-GP100 is a powder polymer designed to modify binders, which has a radial block structure and contains 30% styrene and 70% butadiene by weight.

In addition, sulfur was added at 2.5% with respect to the weight of the SBS polymer. This is composed of 48% cumulative percentage of diluents, dispersants, and conditioners, as well as elemental sulfur (52% by weight) containing particles smaller than 2 μm.

Thus, the product obtained after SBS and sulfur addition corresponded to a modified binder (MB) with a PG 76-22 grade.

### 2.3. Rejuvenating Agent

As the modified binder was to be combined with different dosages of AC-RAP (15%, 30%, and 45% by wt. %), the rejuvenating agent Maro-1000^®^ was used to improve the rheological properties of the new binder while reducing the stiffness caused by oxidation or aging of the AC-RAP. This rejuvenating agent is a viscous yellow-greenish liquid at 25 °C temperature. Its moisture content is 0.314%, its specific density is 0.9546 g/cm^3^, and its Saybolt-Furol viscosity is 292. It has a high inflation point, which does not affect its behavior.

### 2.4. Reclaimed Asphalt Pavement (RAP)

The RAP used in this research’s development was derived by crushing the asphalt binder on the MEX80D “Zapotlanejo-Lagos de Moreno” highway, Jalisco, Mexico. The recovered material was mixed, dried, and homogenized by successive quartering, obtaining representative samples to characterize it.

### 2.5. Experimental Design

**Figure 1:**
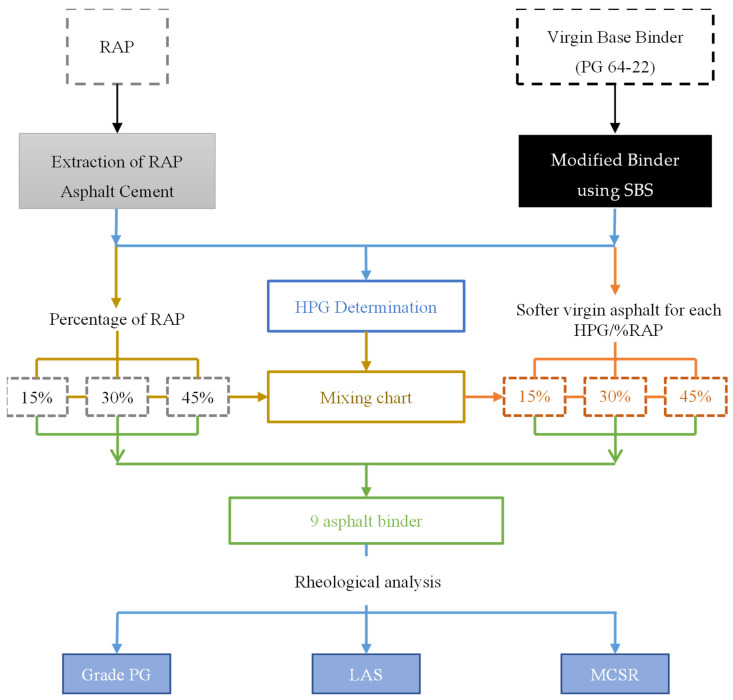
Flow chart of the experimental design.

The experimental design of this article is shown schematically in the flow diagram presented in Figure 1. Preliminarily, it was necessary to obtain a VBB of PG 64-22 grade and RAP, from which AC-RAP was extracted by separating the binder from the aggregate using a solvent–asphalt solution. Following this, a type I SBS polymer was added to the VBB binder, obtaining a modified binder (MB), and three RAP percentages were selected: 15%, 30%, and 45%. Subsequently, the high-temperature performance grade (HPG) of the AC-RAP and modified binder (MB) was obtained to check their PG. The PG of the softened binder corresponding to each percentage of RAP was calculated using a blending chart, considering the HPG of AC RAP and MB and the percentage of RAP with which it was proposed to be combined.

Likewise, AC-RAP, VBB, MB, and asphalt binder were obtained for the low RAP rate (L), medium rate (M), and high rate (H), as well as the combination of the three percentages of AC-RAP by mass ratio with their respective softened binder, which resulted in 9 asphalt binders, as described in Table 1. Softened binders comprised combinations of MB and a dose of rejuvenating agent, a function of the HPG required for each RAP rate.

Finally, the rheological properties of the binders were analyzed by rheology tests on a DSR and BBR, while the nonlinear viscoelastic responses of the nine binder products were evaluated using the MSCR and LAS tests.

### 2.6. Aging Process

In order to simulate the aging of the binder contained in the RAP (AC-RAP) during the manufacturing process of the HMA mixture (short-term) and to eliminate any trace of the solvent that could affect the results of the rheological tests, the short-term aging process for the AC-RAP was performed. The binder specimens were maintained for 85 min at a temperature of 163 °C in the rotary thin-film oven (RTFO, James Cox and Sons, Inc., model FMA-A2308, Colfax, CA, USA) according to ASTM D2872 [45]. In addition, to simulate long-term aging, the pressure aging vessel (PAV, Gilson, model PAV3, Butler, PA, USA) was used according to ASTM D6521 [46], which applies a pressure of 2.1 MPa to the asphalt binder for 20 h at a temperature of 100 °C to simulate the aging of the asphalt binder during its service life.

### 2.7. Extraction of AC-RAP

To perform the RAP rheology tests (DSR and BBR), it was necessary to determine the amount of AC-RAP contained in the RAP, as well as to obtain the solvent–binder solution necessary for the recovery of the AC-RAP. Therefore, the procedure used to quantify the asphalt binder content was carried out in accordance with ASTM D2172 [47] “Standard Test Methods for Quantitative Extraction of Bitumen From Bituminous Paving Mixtures”. In this sense, to estimate the solvent–binder solution, trichloroethylene was used as a solvent to remove the film of the asphalt binder that adhered to the aggregate. Prior to the extraction of the AC-RAP, to make the washing of the mixture more efficient, the RAP material was softened by a preheating process in a 60 °C oven.

Finally, after performing the above procedure repeatedly, a sufficient amount of the solvent–binder solution was obtained. Subsequently, the AC-RAP was recovered through distillation by the Abson method, which follows the standardized procedure described in ASTM D1856 [48] “Standard Test Method for Recovery of Asphalt From Solution by Abson Method”.

### 2.8. Rheological Test

The PG classification of VBB, MB, and AC-RAP binders was performed by rheology tests on a DSR and BBR. Critical failures at high and low temperatures were characterized using the procedures described in AASHTO T315 [49] “Standard Test Method for Determining the Rheological Properties of Asphalt Binder Using a Dynamic Shear Rheometer” and ASTM D6648 [50] and “Standard Test Method for Determining the Flexural Creep Stiffness of Asphalt Binder Using the Bending Beam Rheometer (BBR)”, respectively.

#### 2.8.1. Dynamic Shear Rheometer (DSR)

A dynamic shear rheometer (DSR, Anton Paar, model SmartPave 102e, Styria, Austria) was used to evaluate the rheological properties of bitumen in the domain of linear viscoelastic behavior under different conditions (frequency of 10 rad/s, temperatures of 20–112 °C). The binder samples were tested between parallel plates with a spacing of 1 mm and a plate diameter of 25 mm. The complex shear modulus (G*), phase angle (δ), and rutting factor (G*/sinδ) of the control (unaged) and aged bitumen samples were investigated according to the standard AASHTO T315 [49].

#### 2.8.2. Bending Beam Rheometer (BBR)

The bending beam rheometer (BBSR, ATS, model BBR-2S, Butler, PA, USA) was used to determine the minimum temperature at which the asphalt binder can withstand thermal cracking. The test consists of fabricating a beam of asphalt binder after long-term aging (PAV). The asphalt binder beam was subjected to a load of 0.98 N for 60 s; the temperatures used ranged from 0 to −18 °C. The low failure temperature was determined when the stiffness (St) was 300 MPa and the slope (m-value) of deformation with respect to time was 0.3.

### 2.9. Selection of Properties for Softened Binders

The selection of the binder was based on the HPG grade, calculated using mixing charts governed by Equation (1) [51].

(1) $$\mathrm{Tv}=\frac{\mathrm{Tc}-\left({\%\mathrm{RAP}*\mathrm{Tc}}_{\mathrm{RAP}}\right)}{1-\%\mathrm{RAP}},$$

where Tv = the target failure temperature for the filler binder; that is, the HPG of the project binder plus the rejuvenating additive; Tc = the failure temperature of the project binder (in this case, MB); % RAP = the percentage of the RAP that is to be incorporated into the recycled asphalt mix; Tc_RAP_ = the failure temperature of the aged binder from the RAP (in this case, AC-RAP) after RTFO.

### 2.10. Softened Modified Binder Using a Rejuvenating Agent

**Figure 2:**
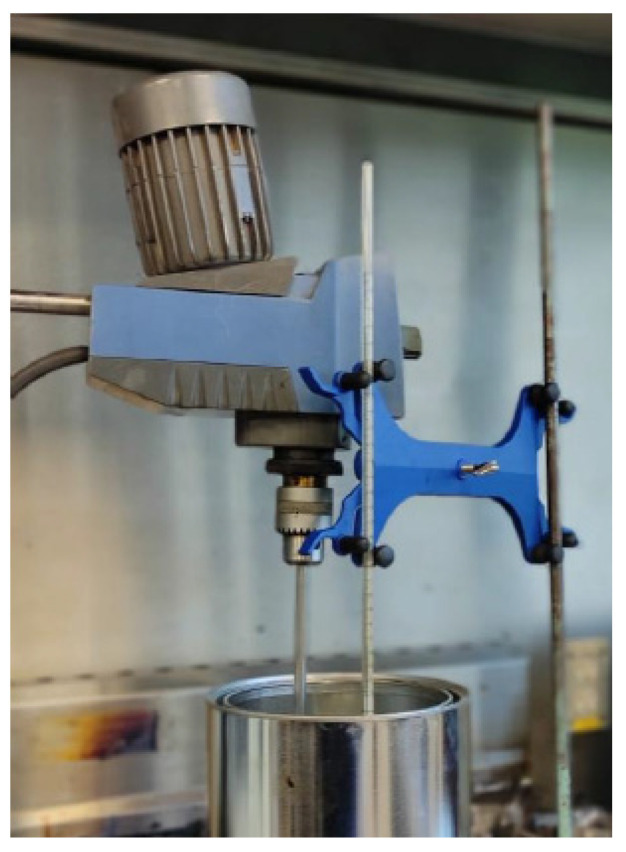
A helix agitator equipped to mix the binder with the SBS polymer, sulfur, and rejuvenating agent.

The use of a softened binder is a technique aimed at counteracting stiffness in the combination of AC-RAP and virgin binder. In this research project, a sufficient volume of modified binder (MB) was obtained and divided into smaller samples, to which different percentages (dosages ranging from 0% to 35%) of the rejuvenating agent, Maro1000^®^, were added. For the homogenization of the MB with different doses of rejuvenating agent, a helix agitator equipped with a digital indicator (IKA Works Inc., model RW20, Wilmington, NC, USA) was used, applying speed rates of 500 and 600 rpm. To guarantee correct homogenization, a vessel containing the binder was preheated in an oven at temperatures in the range of 150–160 °C (see Figure 2).

**Figure 3:**
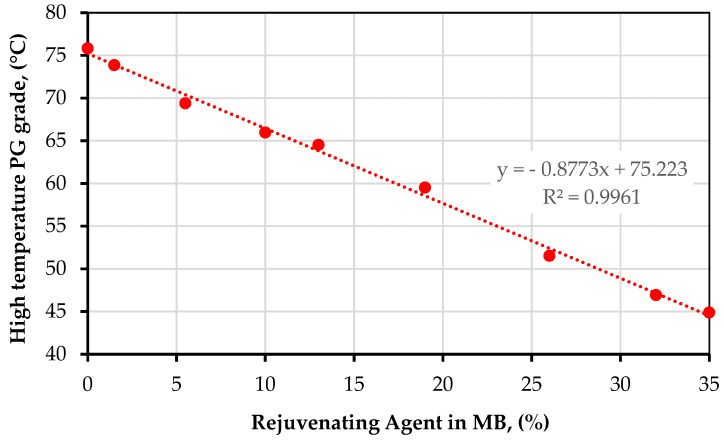
Dependence of HPG on the percentage of rejuvenating agent in MB.

Similarly, the high-temperature PG for each of the samples dosed with rejuvenating agent was obtained. As shown in Figure 3, the percentage of rejuvenating agent in the MB can be estimated, since the HPG decreases linearly with the increase in the rejuvenating agent. With the Tv selection for each RAP rate, it was possible to select the percentage of rejuvenating additive necessary to soften the MB.

### 2.11. Determination of the PG for Softened Binders

Analogous to the characterization of the VBB and MB binders, the softened modified binders with rejuvenating agent (at doses established for each RAP rate) were subjected to DSR and BBR rheological analyses to determine the PG at high and low temperatures.

Thus, softened modified binders with the rejuvenating agent (SMBL, SMBM, and SMBH) and softened modified binders combined with their respective percentages of AC-RAP (SMBL15, SMBM30, and SMBH45) were tested with a DSR before and after RTFO at 163 °C for 85 min. Subsequently, long-term aging was performed with a pressure aging vessel (PAV) test for a re-analysis using the DSR and BBR. The tests were used to determine the permanent deformation parameter (G*/sinδ) and to characterize the PG binders.

### 2.12. Evaluation of the Mechanical Properties of Binders

As the main part of the study, the nonlinear viscoelastic responses of the nine resulting binder products (AC-RAP, VBB, MB, SMBL, SMBM, SMBH, SMBL15, SMBM30, and SMBH45) were evaluated. To determine the effects of the combination of binder, SBS polymer, rejuvenating agent, and AC-RAP, MSCR and LAS tests were performed. The test parameters considered load stresses, corresponding to high levels of stress and accumulated damage, to reference the mechanical behaviors of the binders within the HMA.

#### 2.12.1. Multiple-Stress Creep Recovery (MSCR)

The MSCR test evaluates the mechanical properties of a binder at high temperatures, specifically the performance pertaining to permanent deformation, which correlates to the resistance to deformation accumulated in an asphalt binder [52]. The test procedure used in this research is based on the standardized test specified in AASHTO TP70 [53] “Standard Test Method for the Asphalt Binder MSCR Test Using a DSR”. The MSCR test was performed on samples aged in the RTFO, which had a 25 mm diameter and 1-mm-thick geometric plate. The DSR analyzer applied a shear wave (creep) to the sample for 1 s and then stopped the application of shearing to allow the binder to recover freely during a 9 s rest period (recovery). The 10 s period forms a creep and recovery cycle. The test consists of the application of 10 cycles at stress levels of 0.1 kPa (low force) and 3.2 kPa (high force). The parameters used in the test are shown in Table 2.

As a result of the test, the nonrecoverable creep compliance (Jnr) and the elastic response (%RE) values were obtained, and both parameters were correlated with the rutting performance. This analysis aimed to determine whether the rejuvenating agent dosage was counterproductive to binder performance.

The nonrecoverable creep compliance is a measure of permanent strain and is defined as the percentage of residual unit strain (%ε) of a specimen after being subjected to a loading and recovery cycle divided by the applied stress. The mathematical expression of Jnr is given by Equation (2) [54]:

(2) $$\mathrm{Jnr}=\frac{\mathrm{Non}\text{—}\mathrm{recoverable}\;\mathrm{strain}}{\;\mathrm{stress}\;\mathrm{level}},$$

On the other hand, the %RE represents the recovery of the asphalt binder in the recovery time. The calculation is given by Equation (3):

(3) $$\%\mathrm{RE}=\frac{\mathrm{Recoverable}\;\mathrm{strain}}{\mathrm{Total}\;\mathrm{shears}\;\mathrm{train}}$$

The results reported in this investigation concerning the MSCR test correspond to the Jnr and %RE values calculated in each of the ten cycles for each applied stress level (0.1 kPa and 3.2 kPa).

#### 2.12.2. Linear Amplitude Sweep (LAS)

In this paper, an LAS test was performed based on the standardized test in AASHTO TP101 [55] “Standard Method of Test for Estimating Fatigue Resistance of Asphalt Binders Using the Linear Amplitude Sweep”. A geometry of parallel plates of 8 mm diameter and 2 mm thickness was used.

For the purpose of obtaining comparable results, the temperature selection criterion was taken into account according to the work of van Rooijen, Planque, Wegmann, Di Benedetto, Mangiafico, Sauzeat, Olard and Pouget [44]. Fatigue tests for asphalt binders must be tested at a temperature such that the G* parameter is between 10 MPa and 50 MPa, as measured in the DSR test at 10 Hz and 0.1% strain, within the linear viscoelastic range. The test temperatures for each binder are presented in Table 3.

The test consists of two phases for the same sample. First, a frequency sweep is performed at a constant strain level of 0.1%. The application of load by oscillatory shear stress is performed at frequencies of 0.2, 0.4, 0.6, 0.8, 1, 2, 4, 6, 8, 10, 20, and 30 Hz. The dynamic shear modulus (G*) and phase angle (δ) are recorded at each frequency.

During the second stage, a linear amplitude sweep is performed. Subsequently, the sweep is conducted under the temperature criteria shown in Table 2 at a constant frequency of 10 Hz according to AASHTO TP101 [55]. The stress application is transmitted in 10 s intervals of amplitude under constant strain. Each interval is constituted by 100 load cycles, while the strain amplitude in the first interval is 0.1%. Thus, the strain amplitudes range from 1% to 30%, with an increase of 1% at each point.

The asphalt binder performance can be calculated using the simplified viscoelastic continuous damage theory (S-VECD). Equation (4) shows the potential model [56], based on the peak shear stress experienced during the LAS test.

(4) $$Nf=A{\left(\gamma_{\max}\right)}^{-B}$$

where Nf = the number of cycles to failure; γ_max_ = the maximum strain due to expected tension in the binder under vehicular load; A and B = the coefficients of the VECD model depending on the characteristics of the material. Details of the formulations of coefficients A and B can be found in [57].

The coefficient A represents the ability of the asphalt to maintain its integrity during stress cycles and when under accumulated damage. Meanwhile, coefficient B describes the sensitivity of the asphalt to changes in the level of applied strain. High values of coefficient B indicate that the fatigue life decreases at a faster rate as the amplitude of the strain level increases. Usually, higher A values and lower absolute B values are associated with binders with higher fatigue resistance [58]. The A value indicates the ordinate to the origin and the B value the slope in the ratio of applied stress strain and the number of load cycles (Nf/ESALs). Therefore, a binder with a high value of the A coefficient and a low value of the B coefficient has higher resistance to cracking.

To compare the fatigue resistance between the different binders used and their influence on the mechanical performance of the mixtures that were part of it, it was necessary to calculate by means of the fatigue laws the number of cycles necessary for failure when maximum strains of 2.5% and 5.0% occurred in the binders. The above criteria correspond to the level of deformation that could be expected in binders that are part of strong pavement structures with asphalt layer thicknesses greater than 4 inches (2.5% γ_max_ ≈ 500 μs (εt)) and weak pavement structures with asphalt layers less than 4 inches thick (5.0% γ_max_ ≈ 1000 μs (εt)) [59].

## 3. Results and Discussion

The binders analyzed in this research were based on a virgin base binder (VBB) and aged asphalt (AC-RAP). First, through the addition of SBS-PG100 polymer at a ratio of 2.25% by weight of VBB plus 2.5% sulfur by weight of SBS-PG100 polymer, the VBB binders were modified (MB). Therefore, the rheological properties of VBB, MB, and AC-RAP aged at short term (RTFO) and long term (PAV) were assessed through BBR and DSR rheometry to find the viscoelastic responses of asphalt binders in their initial conditions.

### 3.1. Rheological Properties of Binders

Table 4 shows the rheological properties of VBB, MB, and AC-RAP aged at short (RTFO) and long term (PAV) through BBR rheometry.

From Table 4, it is noticeable that the three binders VBB, MB, and AC-RAP adequately satisfy the specifications required in AASHTO M320 [60]. Considering this result, in Table 4 the G*/sinδ, G*sinδ, St, and m values (related to stiffness) of the AC-RAP binder are shown with respect to the new binders without polymer (VBB) and with polymer (MB). The addition of SBS polymer and sulfur favors elasticity in the binder structure, increasing its viscosity and elasticity. However, the quality of recycled mixes depends not only on the use of new asphalts without or with polymer, but also on the type and dosage of the rejuvenator, meaning overdose of the rejuvenator should be avoided, as this will cause loss of adhesion and detachment of the binder from the aggregate, as established by Devulapalli, Kothandaraman and Sarang [8].

The results in Table 4 show that the maximum temperature obtained from the G*/sinδ parameter in the original binder after RTFO aging for the binder recovered from the RAP (AC-RAP) is 112 °C, which is too high compared to the new binders such as asphalt without polymer (VBB) and polymer-modified asphalt (MB). This shows that the AC-RAP binder is stiffer due to aging during its service life.

On the other hand, the value of G*sinδ is the parameter used to evaluate the behavior at medium temperature at which fatigue cracking occurs. It can be observed that the minimum temperature reached by the binder recovered from the RAP (AC-RAP) after long-term aging using the PAV equipment is 37.3 °C, which is a high temperature compared to the temperatures of the new asphalts without polymer and with polymer (VBB and MB, respectively). This means that any temperature below 37.3 °C at which the AC-RAP test is performed will not satisfy the maximum permissible G*sinδ value of 5000 kPa. This shows how stiff and brittle the AC-RAP binder is due to aging during its service life.

The St value of AC-RAP (temp = 0 °C) increases by 197.24% compared to that of VBB (temp = −12 °C), meaning that the AC-RAP binder is hardened, becoming more rigid and brittle. This was because the binder was exposed to atmospheric conditions (temperature, ultraviolet light, rain, and oxygen), which lead to aging, resulting in the loss of its rheological properties and converting it into a considerably more viscous (stiff) material.

An additional criterion of the BBR test is the m value, which represents the tangent of the creep curve at 60 s time vs. load and indicates the capacity of the binder to dissipate stresses or relax. According to the minimum failure temperatures for the VBB binder of −12 °C and AC-RAP of 0 °C, the m-value of MB is slightly higher than that for VBB, showing a slightly steeper time vs. load curve, indicating that it can resist cracking for longer.

### 3.2. Critical Temperatures in Asphalt Binders

After characterizing the MB binder, it can be seen in Table 4 that the test temperature was 76 °C, thereby determining that the PG grade of the project binder was PG 76-22.

On the other hand, the binder coming from the RAP was extracted, and a sufficient portion of AC-RAP was obtained. Thus, upon verifying that there were no more residues of the solvent used for its separation from the binder with the mineral aggregate, a sample of the AC-RAP binder was analyzed using DSR after undergoing short-term aging in RTFO. The results showed that the critical failure temperature (Tc_RAP_) of AC-RAP was 117.2 °C, whereas the minimum temperature was considered to be the most unfavorable (0 °C).

Although the Superpave methodology does not provide reference parameters for aged asphalts, the theoretical classification of AC-RAP corresponds to PG 112-10. This is the temperature range where the strain-cracking performance is sufficiently capable of resisting rutting and cracking.

Therefore, it can be confirmed that the aging of AC-RAP reflected in Tc_RAP_ is an indicator of brittleness and stiffness compared to that exhibited by the VBB or MB. It is remarkable that by using only AC-RAP, the behavior of AC-RAP results in adequate rutting resistance; however, this implies a high susceptibility to cracking along the asphalt pavement.

### 3.3. Selection of PG for Softened Binders

The stiffness contributed by the RAP is a problem in terms of durability. Therefore, the use of softened binders is an opportunity to counteract the effect shown by AC-RAP. In fact, in the present research, a VBB was used and modified, obtaining a project MB. The MB showed Tc = 76.1 °C, while AC-RAP showed Tc_RAP_ = 117.2 °C, corresponding to HPGs of 76-22 and 112-10, respectively.

To obtain the target temperature (Tv) for the binders SMBL, SMBM, and SMBH (using RAP at 15%, 30%, and 45%, respectively), Equation (1) was employed. In Table 5, it can be observed that the SMBH must be a binder with a grade of PG 40-40; that is, a softer binder than the PG of the SMBL, which requires a 64-28 binder. This makes sense, as the more RAP the mix contains, the stiffer it will be, so it will need a binder soft enough to counteract it.

From the information shown in Table 5, the critical failure temperature (Tc) for high temperatures requires the maximum dosage of rejuvenating agent, taking care not to compromise performance in terms of rutting. On the other hand, the cracking resistance governed by the BBR test requires less of the rejuvenating agent. Therefore, the Tc for high temperatures was the value set for the softening of the binder. Thus, Figure 3 shows that by varying the percentage of rejuvenating agent in an MB sample, a quasi-linear trend can be observed, obtaining the appropriate dosages for the HPGs of SMBL, SMBM, and SMBH.

The percentages of rejuvenating agent calculated based on the mixing chart and Figure 3 are shown in Table 6. The PG grades of the SMBL, SMBM, and SMBH binders were calculated analogously to the VBB and MB binders. From the above, it can be verified that the higher the recycling rate is, the greater the percentage of rejuvenating agent required and the lower the PG, as shown in Table 6. Then, by looking at Table 6, it is evident that SMBH with PG 40-40 mixed with 45% RAP requires 36% rejuvenating agent to soften it.

The softened binders SMBL, SMBM, and SMBH presented significant reductions in the St values of 25.77%, 59.36%, and 89.31%, respectively, when compared to that shown by the MB binder. This provides evidence that the rejuvenating agent improved the rheology and viscosity of the binders, i.e., they were less viscous, which caused a softening of the binders, indicating that they were significantly more flexible than MB.

Similarly, the m-values increased by 10.23%, 151.49%, and 200.33% in comparison with the MB binder. Therefore, these values are considered acceptable, and it is expected that they will continue to comply with required the specifications of AASHTO M320 [60]. This is desirable, since the stiffness of the mix is counteracted, favoring flexibility without compromising fatigue life or decreasing susceptibility to thermal cracking. The requirements of the mixing chart are met to this extent.

### 3.4. Evaluation of the Rheological Properties of Binders

To validate compliance with the mixing charts, the softened binders were combined with the percentage of AC-RAP corresponding to each RAP rate, resulting in the combination of 85% SMBL containing 15% AC-RAP (SMBL15), 70% SMBM containing 30% AC-RAP (SMBM30), and 55% SMBH containing 45% AC-RAP (SMBH45). Additionally, to achieve a part of the objective of this research, the rutting resistance was evaluated through the MSCR test, while the LAS test was used to evaluate fatigue resistance. In this way, high stress levels were applied to assess the accumulated damage using the S-VECD method.

#### 3.4.1. Nonrecoverable Creep Compliance (Jnr) Using MCSR

**Figure 4:**
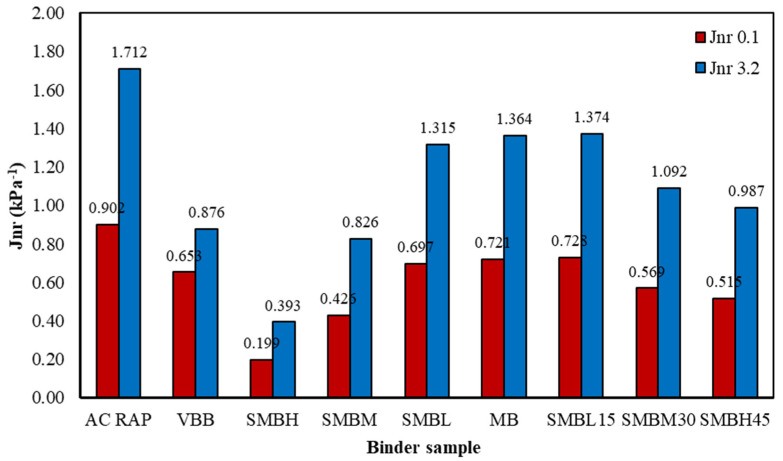
Noncompliance (Jnr) evaluations for each binder.

The nonlinear response of the binders shown through the values Jnr 3.2 kPa^−1^ and Jnr 0.1 kPa^−1^, and the percent elastic response (%RE) were measured in the MSCR test. Both parameters were determined by tests performed at HPG temperature. The Jnr results are summarized in Table 7 and Figure 4.

Figure 4 and Table 7 show that the MB binder (reference binder) presents a value of Jnr_3.2_ = 1.364 kPa^−1^. If this value is compared with those obtained in softened binders containing the rejuvenating agent (SMBL, SMBM, and SMBH), reductions of 3.59%, 39.44%, and 71.19%, respectively, can be observed. Similarly, when analyzing the values of softened binders combined with AC-RAP (SMBL15, SMBM30, and SMBH45), a variation in the Jnr_3.2_ value can also be observed, but at a lower magnitude, i.e., the SMBL15 binder shows an increase of 0.73%, while the SMBM30 and SMBH45 binders present reductions of 19.94% and 27.64%, respectively. This means that the softened binders are softer (more flexible) than MB, being more prone to rutting, even though they adequately comply with the specifications shown in Table 6. In addition, when analyzing the Jnr_0.1_ value, their behavior is very similar to that of the Jnr_3.2_ value.

In contrast, the softened binders blended with AC-RAP (SMBL15, SMBM30, and SMBH45) due to AC-RAP aging were hardened (stiffened), but the addition of the rejuvenating agent softened them, renewing the rheological properties of the binder and counteracting the aging of AC-RAP. The above made it possible to establish a balance by improving the crack resistance without compromising the rutting.

The above is evident when the %RE values are analyzed, i.e., the %RE values of the softened binders with the rejuvenator (SMBL, SMBM, and SMBH) show increases with respect to MB of 14.13%, 32.17%, and 68.04% at 0.1 kPa^−1^, while at 3.2 kPa^−1^ the increases are 29.02%, 77.72%, and 212.44%, respectively. This indicates that the rejuvenator has softened and mitigated the stiffness of the binders, causing them to be significantly softer and prone to permanent strain, yet benefiting cracking behavior.

Analogously, observing the %RE of the binders softened with the rejuvenator blended with AC-RAP (SMBL15, SMBM30, and SMBH45), it can be observed that these also undergo increases of a lesser magnitude, being 9.13%, 16.52%, and 28.485 under 0.1 kPa^−1^, respectively. Likewise, at 3.2 kPa^−1^, the %RE values of these same binders are 15.54%, 36.27%, and 89.64%, respectively. This shows that softened binders blended with AC-RAP are hardened and stiffened by the aging of the AC-RAP, but when the rejuvenating agent is added the rheological properties are restored, thereby softening the binder. This shows that SMBL15, SMBM30, and SMBH45 binders exhibit a more balanced behavior, improving the rutting and fatigue resistance. This coincides with Devulapalli, Kothandaraman and Sarang [8], who concluded that the optimum dose for a rejuvenator is required to produce a stable mixture (with moderate flexibility and stiffness). Additionally, this is consistent with the results described by Baghaee Moghaddam and Baaj [10]. How much a rejuvenator affects or benefits the mixture is not known, meaning proper dosing will prevent overdosing of the rejuvenator, which will cause permanent strains such as rutting, while underdosing will induce cracking.

In addition, the results in Table 7 show that the behaviors of SMBL15 and MB binders (which are of HPG 64 grade) are very similar, indicating that the low percentage of AC-RAP does not significantly modify the behavior of the binder. However, the SBS polymer behavior is mostly manifested when the stress solicitation is higher (3.2 kPa^−1^), while at low stresses (0.1 kPa^−1^) the mechanism of the polymer indicates that at the end of ten loading cycles, the SMBL has a recovery of double its original form, as compared to the VBB having unaltered properties, as obtained from the refinery.

Additionally, it is important to note that the softened binders blended with AC-RAP do not show behavior similar to MB, which is due to the amount of AC-RAP, i.e., the higher the AC-RAP content in the mixture, the more difficult it is to soften it with a rejuvenating agent, as Wang, Zeng, Qin, Huang and Xu [36] explained.

It is important to mention that the Jnr measurement was performed using a standardized method and under controlled conditions, as shown in Table 7; hence, it is reasonable to assume that the distribution of the Jnr results does not change, which implies that they remain practically the same for measurements performed under the same conditions [61]. In this case, this component of the uncertainty can be more reliably estimated with the standard deviation obtained from a single experiment than with the experimental standard deviation derived from n number of measurements. The Jnr results for 0.1 kPa^−1^ present a standard deviation of 0.204 kPa^−1^ and a standard error of 0.068 kPa^−1^, while for Jnr 3.2 kPa^−1^ the standard deviation and standard error are 0.387 and 0.129 kPa^−1^, respectively.

**Figure 5:**
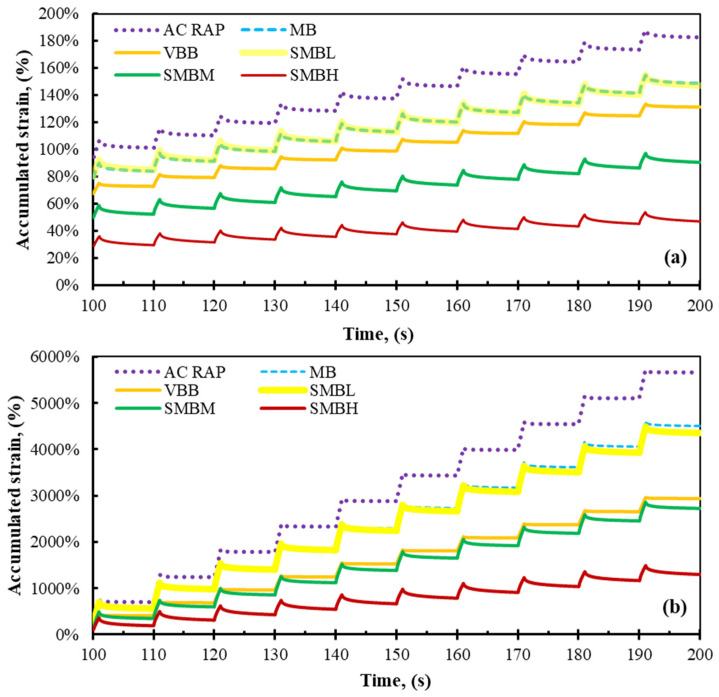
Accumulated strain values of softened modified binders at (**a**) 0.1 kPa^−1^ and (**b**) 3.2 kPa^−1^.

**Figure 6:**
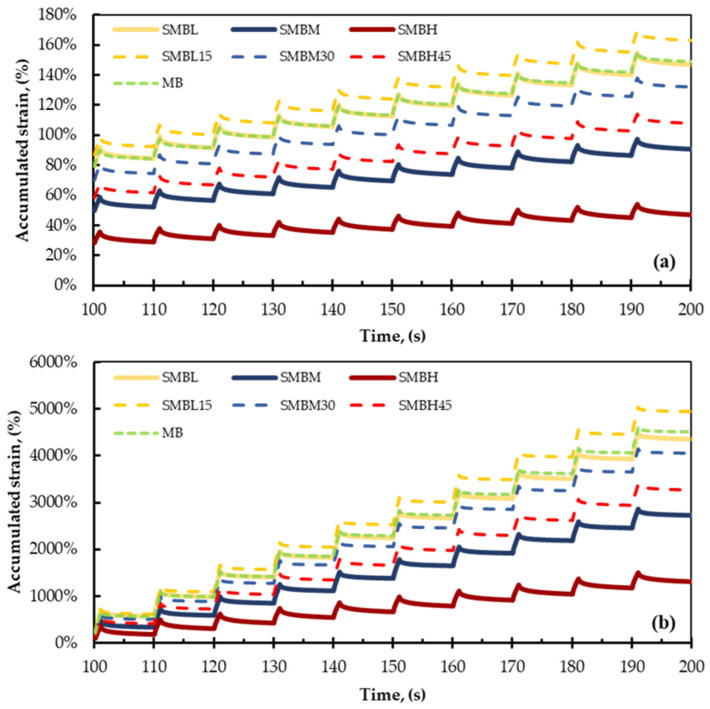
Accumulated strain values of binders blended with AC-RAP vs. softened modified binders at (**a**) 0.1 kPa and (**b**) 3.2 kPa.

Observing Figure 5a,b and Figure 6a,b, and Table 7, it is evident that the relationship between the Jnr values and the accumulated strain is significant; in other words, the higher the Jnr value, the greater the accumulated strain, and vice versa.

Therefore, analyzing the total accumulated strain shown in Figure 5a,b, corresponding to softened binders with SBS polymer subjected to stresses of 0.1 kPa^−1^ and 3.2 kPa^−1^, respectively, it can be observed that the AC-RAP binder shows the highest levels of accumulated strain in each of the loading cycles. This result is consistent with Table 7, since AC-RAP presents the most unfavorable Jnr and RE% values, indicating that it suffers more rutting damage, although this behavior is not appropriate for an aged binder, due to having the highest test temperature (112 °C). Similarly, comparing the MB and SMBL binders, they show similar behavior, manifesting minimal differences despite the variation in test temperature (76 and 64 °C, respectively).

The VBB and the binders softened with SBS polymer (SMBM and SMBH) show the best rutting performance (atypical behavior of softened binders), but since the test temperature is the lowest of all the tests (64, 58, and 40 °C, respectively), they are stiffer and prone to fatigue cracking.

Analogously, in Figure 6a,b, it can be seen that the MB and SMBL binders behave similarly, as observed in Figure 5a,b. However, when 15% AC-RAP is added, SMBL15 shows a decrease in rutting resistance.

When analyzing the softened binders blended with AC-RAP (SMBM30 and SMBH45), it can be observed that they perform better against rutting compared to MB, SMBL, and SMBL15 binders. In contrast, SMBM30 and SMBH45 binders show better fatigue cracking performance compared to SMBM and SMBH. Thus, in this analysis, it can be observed that binders with a low percentage of AC-RAP (15%) do not present significant variation with respect to virgin binders, while the addition of AC-RAP in higher proportions (30% and 45%) results in binders with balanced rutting and fracture cracking resistance.

However, due to differences in test temperatures, the binders exhibit inconsistent behavior. Therefore, future work will seek to evaluate the rheological properties and mechanical behavior of softened binders with different polymers, blended with AC-RAP (using different proportions), and using similar test temperatures, in order to characterize the binders using SEM and XRD techniques.

#### 3.4.2. LAS Test Results

The fatigue phenomenon was calculated using the simplified viscoelastic continuum damage (S-VECD) theory; this model’s input variables depend on the results obtained in the LAS test. However, the current test protocol does not specify a test temperature for binders that have characteristics similar to those analyzed in this paper. Temperature is a relevant factor in evaluating the viscoelastic behavior and mechanical performance of an asphalt binder [62]. Therefore, the materials were tested at the same stiffness (G*), ensuring the cohesive failure of fatigue cracking in the specimen. The complex shear modulus parameter was set at 10 ≤ G* ≤ 50 MPa to meet the viscoelastic solid condition, where δ < 45°.

The previous criterion was based on the performance of the binder, avoiding temperatures that could underestimate or overestimate the performance of the analyzed binders.

**Figure 7:**
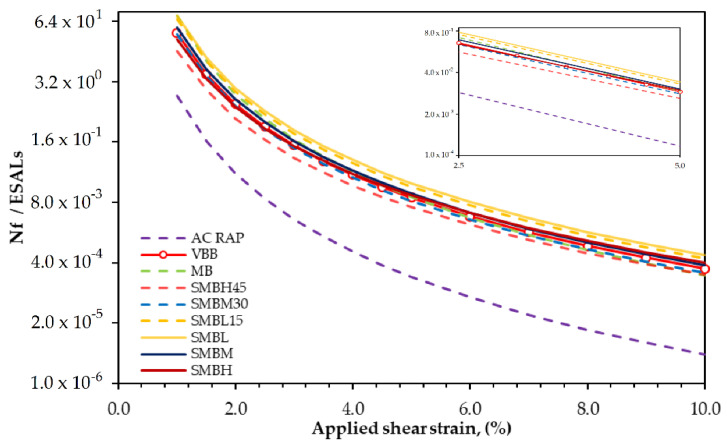
Number of cycles to failure versus pavement strain for original, aged, and modified asphalt binder.

The LAS test relates the behavior of asphalts to fatigue cracking. Figure 7, derived from the LAS test, shows the relationship between the number of stress cycles through a viscoelastic model (Equation (4)), whereby the binder withstands failure with respect to the percentage of maximum applied shear strain.

From Equation (4) and Figure 7, it can be observed that a higher A value (ordinate at the origin) is related to the resistance to cracking, where it can be observed that the AC-RAP binder presents the most unfavorable behavior due to aging during its service life. The highest value of this parameter is presented by the softened binders (SMBL and SMBL15).

The parameter B (slope) is related to the binder’s susceptibility to cracking due to changes in applied shear stress. Thus, AC-RAP is the binder with the highest value of B, while the softened binders SMBL, SMBM, and SMBH have the lowest values.

Additionally, by analyzing the load cycles to failure (Nf/ESALs) of the binders under an applied shear strain range of 2.5 to 5.0%, which represents the interval that relates the fatigue cracking behavior of an asphalt layer with thicknesses greater than 4 inches and less than 4 inches, respectively, SMBL and SMBL15 binders show the best behavior under both applied shear strains, while AC-RAP shows the lowest values under both strains, being susceptible to fatigue cracking due to age hardening.

In summary, according to the results shown in Figure 7, in the LAS test, it is remarkable that the binders softened with the polymer (MB, SMBL, SMBM, and SMBH) show the best cracking behavior, while, when blended with a percentage of AC-RAP (SMBM30 and SMBH45) higher than 15%, they are more prone to cracking. SMBL and SMBL15 binders again show similar behaviors. Finally, the LAS test again shows that SMBM30 and SMBH45 binders have moderate rutting and fatigue cracking resistance. Additionally, it is notable that as the percentage of AC-RAP increases, it is more difficult to soften the binder and recover its rheological properties.

## 4. Conclusions

The objective of this work was to analyze the behavior of different binders, including virgin binder (VBB), binder modified by SBS polymer (MB), AC-RAP, binder softened using a rejuvenating agent, and binders softened containing doses (15%, 30%, and 45%) of AC-RAP. The rheological properties were evaluated by dynamic shear rheometry (DSR) and beam-bending rheometry (BBR) tests. To verify the integration and rejuvenation of the binder, as well as the proportion of rejuvenating agent needed to soften each AC-RAP content was obtained from the mixing chart. Thus, the linear amplitude sweep (LAS) test was used to measure fatigue cracking and the multiple stress creep recovery (MSCR) test to measure rutting. The frequency sweep accelerated the damage accumulation rate using the continuous viscoelastic damage theory (VECD).

From the rheological tests, it was observed that the VBB, MB, AC-RAP, SMBL, SMBM, and SMBH binders correctly satisfied the specifications. It was also observed that adding a rejuvenating agent to the VBB resulted in a 16.19% more flexible MB binder due to the addition of the SBS polymer and sulfur, which promoted adhesion within the binder structure, reducing its viscosity and stiffness. AC-RAP increased in stiffness by 197.24% compared to VBB, which meant that it was hardened and brittle, because the AC-RAP was exposed to aging by temperature, ultraviolet light, rain, and oxygen, turning it into a viscous (stiff) material. Additionally, the softened binders (St_SMBL_ = 74.92 MPa, St_SMBL_ = 41.02 MPa, and St_SMBL_ = 10.79 MPa) showed decreased stiffness (25.77%, 59.36%, and 89.31%) compared to the MB binder (St_MB_ = 100.93 MPa). This shows that the rejuvenating agent improved the rheology and viscosity of the binders, making them less viscous, softer, and significantly more flexible than MB. Therefore, an optimum dose of rejuvenator is required to produce a stable mixture (with adequate flexibility and stiffness).

After characterizing the MB binder, the test temperature was 76 °C, thereby determining that the PG of the project binder was PG 76-22, while the theoretical classification of AC-RAP corresponded to PG 112-10 because the critical failure temperature (TC_RAP_) of AC-RAP was 117.2 °C and the minimum temperature was the most unfavorable (0 °C). Therefore, it can be confirmed that the aging of AC-RAP reflected in TC_RAP_ is an indicator of brittleness and stiffness compared to that exhibited by the VBB or MB.

The MB binder (reference binder) presented a value of Jnr_3.2_ = 1.364 kPa^−1^. If this value is compared with those obtained in softened binders (Jnr_3.2SMBL_ = 1.315 kPa^−1^, Jnr_3.2SMBM_ = 0.826 kPa^−1^, and Jnr_3.2SMBH_ = 0.393 kPa^−1^), reductions of 3.59%, 39.44%, and 71.19%, respectively, can be observed. Analogously, comparing the Jnr_3.2_ values of softened binders blended whit AC-RAP (Jnr_3.2SMBL15_ = 1.374 kPa^−1^, Jnr_3.2SMBM30_ = 1.092 kPa^−1^, and Jnr_3.2SMBH45_ = 0.987 kPa^−1^), the SMBL15 binder shows an increase of 0.73%, while the SMBM30 and SMBH45 binders present reductions of 19.94% and 27.64%, respectively. The MCSR tests show that the softened binders are softer (more flexible) than MB, being more prone to rutting, while softened binders blended with AC-RAP are hardened by RAP aging, although the addition of the rejuvenating agent softens them, renewing the rheological properties of the binder and counteracting the aging of AC-RAP. Thus, the softened binders blended with AC-RAP show a balanced behavior by improving the crack resistance without compromising rutting.

From the LAS test, it can be observed that the binders softened with the polymer (MB, SMBL, SMBM, and SMBH) show the best cracking behavior, but when blended with a percentage of AC-RAP (SMBM30 and SMBH45) higher than 15%, they are more prone to cracking due to RAP aging. The SMBL and SMBL15 binders again show similar behaviors to MB, indicating that by adding a low percentage (15%) of AC-RAP, the fracture behavior is not significantly affected. As demonstrated in this analysis, the AC-RAP should be added with a rejuvenating agent to recover its flexibility and rheological resistance, since it will improve the cohesion, adhesion, and resistance to fatigue cracking in the asphalt mixture.

Due to differences in test temperatures, the binders showed inconsistent behavior throughout the MCSR test. Therefore, future work will attempt to evaluate the rheological properties and mechanical behavior of binders softened with different polymers, blended with AC-RAP (using different ratios), and with similar test temperatures in order to characterize the binders through SEM and XRD techniques.

## Figures and Tables

**Table 1:** Binder matrices and definitions.

Binder Acronym	Definition
AC-RAP	Recovered RAP Asphalt Cement
VBB	Virgin Base Binder
MB	Modified binder with 2.25% SBS
SMBL	Softened Modified Binder using 2.25% SBS and rejuvenating agent for Low-rate RAP
SMBM	Softened Modified Binder using 2.25% SBS and rejuvenating agent for Medium-rate RAP
SMBH	Softened Modified Binder using 2.25% SBS and rejuvenating agent for High-rate RAP

**Table 2:** Parameters used in the MSCR test.

Description	Specification Requirement
Equipment	Dynamic Shear Rheometer (DSR)
Aging	Residue RTFO
Geometry (diameter/thickness)	25 mm/1 mm
Temperature criteria	HPG
MSCR Low Stress	(Creep 0.1 kPa, 1 s/Recovery 9 s) × 10 cycles
MSCR High Stress	(Creep 3.2 kPa, 1 s/Recovery 9 s) × 10 cycles

**Table 3:** Parameters considered in the LAS test.

Description	Specification Requirement
Equipment	Dynamic Shear Rheometer (DSR)
Aging	PAV
Geometry (diameter/thickness)	8 mm/2 mm
Temperature criteria	10 ≤ G* ≤ 50 MPa @10 Hz-0.1% strain
Frequency sweep	Constant strain 0.1%, Frequencies from 0.2 to 30 Hz
Linear Amplitude Sweep	Constant Frequency 10 Hz, Strain from 0 to 30%

**Table 4:** Rheological properties of VBB, MB, and AC-RAP aged for short (RTFO) and long terms (PAV) through BBR rheometry.

Binder Sample	Characteristic	Specification Requirement AASHTO M320 [60]	Temperature °C	Value	%
VBB	Original binder, kPa	G*/Sinδ ≥ 1.0 kPa	64	1.81	-
	RTFO, kPa	G*/Sinδ ≥ 2.2 kPa	64	8.67	-
	PAV, kPa	G*x Sinδ ≥ 5000 kPa	25	2447.5	-
	BBR Stiffness, MPa	St ≤ 300 MPa	−12	120.42	-
	BBR m-value	m-value ≥ 0.300		0.301	-
MB	Original binder, kPa	G*/Sinδ ≥ 1.0 kPa	76	1.07	−40.88
	RTFO, kPa	G*/Sinδ ≥ 2.2 kPa	76	5.07	−41.52
	PAV, kPa	G*x Sinδ ≤ 5000 kPa	31	1365.9	−44.19
	BBR Stiffness, MPa	St ≤ 300 MPa	−12	100.93	−16.19
	BBR m-value	m-value ≥ 0.300		0.303	+0.66
AC-RAP	RTFO, kPa	G*/Sinδ ≥ 2.2 kPa	112	3.67	−57.67
	PAV, kPa	G*x Sinδ @ 5000 kPa	37.3	5000	104.29
	BBR Stiffness, MPa	St @ 300 MPa	0	300	197.24
	BBR m-value	m-value @ 0.300		0.300	−0.99

**Table 5:** Rheological properties shown as a blending chart.

Temperature	Critical Temperature (Tc), °C		Tc Requirement Blending Chart for Each %RAP		
	AC-RAP	MB	15%	30%	45%
High	117.2 1	76.1 1	68.8	58.5	42.47
Medium	37.3 2	16.9 2	13.3	8.2	0.21
Low	−10	−22	−25.88	−31.42	−40
Requirement PG			64-28	58-34	40-40

**Table 6:** Dose of agent rejuvenator and rheological properties of soft binders.

Binder Sample	Req. PG	Rejuve. Agent	Characteristic	Specification Requirement AASHTO M320 [60]	Temperature, °C	Value	%
SMBL	64-28	10%	Original binder, kPa	G*/Sinδ ≥ 1.0 kPa	64	1.35	26.17
			RTFO, kPa	G*/Sinδ ≥ 2.2 kPa	64	4.61	−9.07
			PAV, kPa	G*x Sinδ ≤ 5000 kPa	22	2556.3	287.15
			BBR Stiffness, MPa	St ≤ 300 MPa	−18	74.92	−25.77
			BBR m-value	m-value ≥ 0.300		0.334	10.23
SMBM	58-34	15%	Original binder, kPa	G*/Sinδ ≥ 1.0 kPa	58	1.51	41.12
			RTFO, kPa	G*/Sinδ ≥ 2.2 kPa	58	5.86	15.58
			PAV, kPa	G*x Sinδ ≤ 5000 kPa	16	2396.7	75.47
			BBR Stiffness, MPa	St ≤ 300 MPa	−18	41.02	−59.36
			BBR m-value	m-value ≥ 0.300		0.762	151.49
SMBH	40-40	36%	Original binder, kPa	G*/Sinδ ≥ 1.0 kPa	40	1.76	64.49
			RTFO, kPa	G*/Sinδ ≥ 2.2 kPa	40	7.35	44.97
			PAV, kPa	G*x Sinδ ≤ 5000 kPa	NA	NA	NA
			BBR Stiffness, MPa	St ≤ 300 MPa	−18	10.79	−89.31
			BBR m-value	m-value ≥ 0.300		0.910	200.33

**Table 7:** MSCR test results.

Binder Sample	Temperature, °C	RE_0.1_, %	RE_3.2_, %	Jnr_0.1_, kPa^−1^	Jnr_3.2_, kPa^−1^
VBB	64	0.262	0.080	0.653	0.876
MB	76	0.460	0.193	0.721	1.364
AC-RAP	112	0.369	0.077	0.902	1.712
SMBL	64	0.525	0.249	0.697	1.315
SMBM	58	0.608	0.343	0.426	0.826
SMBH	40	0.773	0.603	0.199	0.393
SMBL15	76	0.502	0.223	0.728	1.374
SMBM30	76	0.536	0.263	0.569	1.092
SMBH45	76	0.591	0.366	0.515	0.987

## Data Availability

Not Applicable.
